# Why do men go to the doctor? Socio-demographic and lifestyle factors associated with healthcare utilisation among a cohort of Australian men

**DOI:** 10.1186/s12889-016-3706-5

**Published:** 2016-10-31

**Authors:** Marisa Schlichthorst, Lena A. Sanci, Jane Pirkis, Matthew J. Spittal, Jane S. Hocking

**Affiliations:** 1 Melbourne School of Population and Global Health, the University of Melbourne, Melbourne, 3010 Australia; 2 Department of General Practice, the University of Melbourne, Melbourne, 3010 Australia

## Abstract

**Background:**

Men use health services less often than women and frequently delay seeking help even if experiencing serious health problems. This may put men at higher risk for developing serious health problems which, in part, may explain men’s higher rates of some serious illnesses and shorter life span relative to women. This paper identifies factors that contribute to health care utilisation in a cohort of Australian men by exploring associations between socio-economic, health and lifestyle factors and the use of general practitioner (GP) services.

**Methods:**

We used data from Ten to Men, the Australian Longitudinal Study on Male Health. Health care utilisation was defined in two ways: at least one GP visit in the past 12 months and having at least yearly health check-ups with a doctor. Associations between these two measures and a range of contextual socio demographic factors (education, location, marital status, country of birth, employment, financial problems etc.) as well as individual health and lifestyle factors (self-rated health, smoking, drinking, healthy weight, pain medication) were examined using logistic regression analysis. The sample included 13,763 adult men aged 18 to 55 years. Analysis was stratified by age (18 to 34 year versus 35 to 55 years).

**Results:**

Overall, 81 % (95 % CI: 80.3–81.6) of men saw a GP for consultation in the 12 months prior to the study. The odds of visiting a GP increased with increasing age (*p* < 0.01), but decreased with increasing remoteness of residence (*p* < 0.01). Older men, smokers and those who rate their health as excellent were less likely to visit a GP in the last 12 months, but those on daily pain medication or with co-morbidities were more likely to have visited a GP. However, these factors were not associated with consulting a GP in the last 12 months among young men.

Overall, 39 % (95 % CI: 38.3–39.9) reported having an annual health check. The odds of having an annual health check increased with increasing age (*p* < 0.01), but showed no association with area of residence (*p* = 0.60). Across both age groups, the odds of a regular health check increased with obesity and daily pain medication, but decreased with harmful levels of alcohol consumption.

**Conclusion:**

The majority of men (61 %) did not engage in regular health check-up visits, representing a missed opportunity for preventative health care discussions. Lower consultation rates may translate into lost opportunities to detect and intervene with problems early and this is where men may be missing out compared to women.

## Background

Numerous studies have found that men overall have lower rates of medical help seeking and health care utilisation compared with women [1–6]. This behaviour has been observed across a diverse range of health problems such as general physical health problems, mental health problems, life-stresses, and alcohol and substance use [7–14]. Data on primary care in the UK show that among those aged 21 to 58 years, men consult a general practitioner (GP) half as often as women [15], a difference that wasn’t explained by women attending for reproductive health reasons. There are also considerable gender differences in terms of health outcomes - life expectancy is greater in women compared with men in most developed countries and men also suffer higher rates of many non-communicable diseases including heart disease, lung cancer, chronic respiratory disease, alcoholism and suicide [2]. It is possible that men’s lower rates of health care utilisation may be contributing to these differences in health outcomes [16].

Primary care provides a crucial role in preventive health care activities including promoting smoking cessation, responsible alcohol consumption, weight control and physical activity and undertaking screening activities including blood pressure monitoring, cholesterol and blood glucose measurement and cancer screening [17]. Any difference in consultation patterns between the sexes could therefore have important implications for future health outcomes particularly given that preventive activities are often conducted opportunistically when the patient presents for other reasons in general practice. Infrequent attendees have been shown to receive less preventive care [18]. Higher consultation rates may therefore translate into greater opportunities to detect and intervene with problems early and this is where men may be missing out compared to women [19]. Once detected however, men and women with common morbidities such as depression and cardiovascular disease show similar patterns of health care utilisation [15].

Patterns of health care utilisation are complex and it is likely that a variety of masculinity ideologies, norms, and gender roles play a part in discouraging men’s help seeking [20]. Most studies investigating health care utilisation compare genders and hence have limited scope to examine factors associated with health care utilisation among men and to explore differences across sub-groups of men [20, 21]. Besides gender, socio-economic status, access to services, cost of services, age, level of education and marital status have been associated with service utilisation [22–26]. Negative attitudes to help seeking have been discussed as a barrier to help seeking in men [20, 27, 28], particularly in the context of mental health problems [26, 29, 30]. Gender specific attitudinal and behavioural factors are also important predictors for medical services utilisation [1]. Over the last decade a growing body of research links men’s reluctance to seek help to adherence to traditional masculine norms (e.g. stoicism) [22, 31–44]. However, these studies lack evidence on the processes driving this association.

Research into male-specific patterns of health care utilisation has been limited by small sample size and/or focus on specific or high prevalence male health issues. To date a comprehensive analysis of social, behavioural and socio-economic factors associated with health care utilisation in men and how this may change over time as men age is missing from the literature. We have the unique opportunity to explore health care utilisation among men in an ongoing cohort study of Australian men aged 18 to 55 years. In this paper, we present the cross-sectional analysis investigating associations with two measures of health care utilisation (past visit to a general practitioner (GP) and regular health check-ups for men) and a number of contextual socio-demographic characteristics and general health and lifestyle choices at recruitment. These data will form the baseline for future analyses in subsequent waves of the data collection of the cohort.

## Methods

### Participants

We used data from Ten to Men (the Australian Longitudinal Study on Male Health) a population-based cohort study capturing a range of health outcomes, health behaviours and related risk factors (including social determinants). The study methodology has been fully described by Currier and colleagues in this supplement of BMC Public Health [45]. Briefly, participants were recruited from households between October 2013 and July 2014 based on a stratified, multi-stage, random cluster sampling design. Regional areas were over-sampled to increase the numbers in these groups and allow for meaningful analysis in these population sub-groups. Men were asked to complete a detailed questionnaire that covered a range of health outcomes, behaviours and related risk factors. The final sample consists of 15,988 male participants aged between 10 and 55 years. For the purpose of this paper, respondents under 18 years of age were excluded from the analysis leaving the total number of participants at 13,763 men. About 60 % of men live in major cities, 23 % in inner regional and 19 % in outer regional areas. Compared with Australian census data, older and middle aged men were slightly over represented with about a third of men each in the 35 to 44 years and the 45 to 55 years age groups, while 18 to 24 year old men were underrepresented at about 15 % of the sample. The majority was born in Australia (77 %), completed year 12 of secondary school (60 %), were married or in a de-facto relationship (67 %), were employed (86 %), and a father to at least one child (66 %).

### Measures

Two questions from the Ten to Men adult questionnaire were used to assess health care utilisation. The first asked participants to name the health services or type of health professional (e.g., dentist, GP, chiropractor etc.) with whom they had consulted in the 12 months prior to the survey. A list of 25 services was provided including an option to name others. Participants marked “yes” to all services that applied to them. The second question asked participants to state how often they saw their family doctor for a general health check-up, i.e. not because they are sick or injured. The response options were more than once a year, once a year, less frequently, and never.

The predictors of health care utilisation we examined included a range of socio-demographic characteristics and health and lifestyle factors. Region of residence follows the Australian Statistical Geographical Standard Classification and was coded into major city, inner regional and outer regional areas [46]. Participants’ age was recoded into four groups: 18 to 24 years, 25 to 34 years, 35 to 44 years and 45 to 55 years. Highest level of secondary school education was aggregated for those who completed year 12 of secondary school versus those who had not completed secondary school. Participants born outside Australia were combined and contrasted against those born in Australia. Due to low numbers of respondents in some categories ‘marital status’ was recoded into three categories: never married, widowed/divorced/separated, and married/de facto. Employment status captured employed versus currently unemployed men. Based on the number of children per participant a new variable was created for being a father (i.e. having at least one child) and not being a father (i.e. having no children). Financial difficulty was assessed using six questions from a national expenditure survey: i.e. not filling or collecting a prescription medicine; not getting a medical test, treatment or follow-up that was recommended by a doctor; limiting fruit or vegetable consumption; not paying for electricity, gas or telephone bills on time; and having to ask friends or family for financial help [47]. We aggregated responses across these items and recoded into a dichotomous variable to identify participants who experienced at least one financial difficulty in the past 12 months. Whether or not the participant had private health insurance was also included as a binary variable.

The health and lifestyle factors we examined were participants’ current smoking status (smoker versus non-smoker), harmful or hazardous alcohol consumption (derived from the Alcohol Use Disorder Identification Test - AUDIT) [48], and obesity (derived from the body mass index (BMI) and categorised as obese with BMI greater or equal 30 and non-obese with BMI less than 30). A new variable was generated to capture participants who used over the counter pain-medication on a daily basis. Finally, self-rated general health was measured using the first question of the SF12 Health Survey [49]. The original 5-point scale was dichotomized into very good-excellent health versus poor-fair-good health. To control for the presence of health conditions, a comorbidity score was derived as an aggregate across 19 high prevalence conditions indicating if the participant was diagnosed with these in the past 12 months. Participants were then grouped into four categories: diagnosed with no health condition in the previous 12 months, diagnosed with one health condition, diagnosed with two health conditions and diagnosed with 3 or more health conditions.

### Statistical analysis

Our primary outcome was consultation with a GP in the last 12 months defined as whether or not the participant had visited a GP for their own health at least once during the last 12 months. Our secondary outcome was whether or not the participant had a regular check-up at least once per year (defined as visiting a GP for a general health check-up, not because he was sick or injured).

Proportions and 95 % confidence intervals (CI) were first calculated to estimate health care use. Trends by age and level of geographic remoteness were assessed using the Score test. We used logistic regression to assess associations between socio-demographic and health and lifestyle factors and the primary and secondary outcomes. In a first step we ran regression analysis accounting for interaction effects between age and other exposures to identify any important interaction. Where the interaction effects were significant we stratified the analysis by age groups. To investigate any bias associated with missing data, the demographic characteristics of those included in the analysis were compared with those excluded. All analysis use unweighted data and were performed using Stata/IC 13.1.

## Results

### Consultation with a GP in the last 12 months

Of the 13,763 participating men, 11,148 (81 %, 95 % CI: 80.3–81.6) had consulted with their GP in the 12 months prior to the study. Participants also reported consulting with a dentist (39 %), specialist doctors (25 %), pharmacist (20 %), optometrist (13 %), chiropractor (14 %), physiotherapists (13 %), and nurses (6.5 %) in the preceding 12 months. Overall, 12,264 men (89 %, 95 % CI: 88.6–89.6) reported consulting with at least one healthcare provider in the last 12 months with participants visiting an average of 2.5 different health services (SD = 2.13, 95 % CI: 2.44–2.51). There was a trend towards increasing odds for visiting a GP in the last 12 months as age increased (*p* < 0.01) and as remoteness levels decreased (*p* < 0.01). A small proportion of men (8 %, 95 % CI: 7.3–8.2) indicated that they were unable to access health care when needed in the last 12 months. For this group, there was no evidence of a trend of increasing or decreasing odds by age (*p* = 0.35), but there was evidence for a trend of increasing odds by remoteness level (*p* < 0.01).

Preliminary regression analysis for GP visits found significant interaction effects between age and health check-up (*p* = 0.006), employment status (*p* = 0.002) and positively self-rated health (*p* = 0.004). For the ease of reporting we stratified the final regression models by age groups.

Multivariate analysis showed that among 18 to 34 years old participants, the odds of consultation with a GP in the last 12 months were increased among those who had regular yearly health check-ups (OR = 4.5, 95 % CI: 3.4–5.9), for those who had been diagnosed with health conditions and was greatest for those with three or more conditions compared with none (OR = 4.4, 95 % CI: 2.2–8.6), for those who completed secondary school (OR = 1.3, 95 % CI: 1.0–1.6), were born in Australia (OR = 1.3, 95 % CI: 1.0–1.7), were in employment (OR = 1.5, 95 % CI: 1.2–1.9), had private health insurance (OR = 1.3, 95 % CI: 1.1–1.6), had experienced financial difficulties in the past 12 months (OR = 1.3, 95 % CI: 1.0–1.3), and those who are a father (OR = 1.4, 95 % CI: 1.1–1.8). The odds of consultation with a GP decreased in this age group with residing outside of a major city (inner regional area OR = 0.7, 95 % CI: 0.5–0.9; outer regional area OR = 0.7, 95 % CI: 0.5–0.9). None of the health and lifestyle factors showed significant associations (Table 1).

**Table 1:** Correlates of health service utilisation – for ‘GP visit in the past 12 months’ and ‘yearly health check-ups’

	Model 1: Correlates of ‘GP visit in past 12 months’				Model 2: Correlates of ‘regular health check-ups’			
	18–34 years old men		35–55 years old men		18–34 years old men		35–55 years old men	
	Univariate regression	Multivariate regression	Univariate regression	Multivariate regression	Univariate regression	Multivariate regression	Univariate regression	Multivariate regression
	Odds ratio [95 % CI]	Odds ratio [95 % CI]	Odds ratio [95 % CI]	Odds ratio [95 % CI]	Odds ratio [95 % CI]	Odds ratio [95 % CI]	Odds ratio [95 % CI]	Odds ratio [95 % CI]
Control variables								
Yearly health check-ups								
At least once per year	4.43 (3.35–5.86)	4.45 (3.35–5.92)	9.02 (7.05–11.52)	7.01 (5.46–9.01)	-	-	-	-
Less often or never	-	-	-	-	-	-	-	-
GP visit in past 12 months								
Yes	-	-	-	-	4.43 (3.35–5.86)	4.40 (3.31–5.85)	9.02 (7.05–11.52)	7.00 (5.45–8.98)
No	-	-	-	-	-	-	-	-
Diagnosis of health conditions in past 12 months								
None	-	-	-	-	-	-	-	-
1 condition	2.30 (1.77–3.00)	2.11 (1.61–2.76)	3.40 (2.72–4.24)	2.57 (2.04–3.24)	1.37 (1.12–1.67)	1.21 (0.99–1.49)	2.13 (1.88–2.42)	1.82 (1.60–2.08)
2 conditions	3.71 (2.36–5.86)	3.36 (2.09–5.38)	5.64 (3.92–8.12)	3.72 (2.55–5.42)	2.06 (1.58–2.68)	1.76 (1.33–2.32)	2.87 (2.44–3.37)	2.37 (2.00–2.81)
3 or more conditions	4.23 (2.21–8.10)	4.37 (2.23–8.56)	6.23 (4.10–9.45)	3.17 (2.03–4.94)	1.73 (1.21–2.47)	1.39 (0.95–2.03)	3.90 (3.26–4.66)	3.08 (2.52–3.77)
Socio-demographic characteristics								
Remoteness								
Major City	-	-	-	-	-	-	-	-
Inner Regional	0.72 (0.58–0.88)	0.68 (0.54–0.85)	0.73 (0.62–0.88)	0.77 (0.64–0.94)	0.95 (0.78–1.15)	1.05 (0.86–1.28)	0.95 (0.84–1.08)	0.97 (0.84–1.11)
Outer Regional	0.69 (0.55–0.86)	0.67 (0.53–0.85)	0.89 (0.73–1.08)	0.92 (0.74–1.13)	0.96 (0.78–1.18)	1.09 (0.87–1.36)	0.97 (0.85–1.11)	0.96 (0.83–1.11)
Education								
Completed Year12	1.23 (1.02–1.49)	1.25 (1.00–1.55)	1.07 (0.92–1.24)	1.16 (0.97–1.37)	1.08 (0.90–1.29)	0.98 (0.81–1.20)	0.78 (0.70–0.86)	0.73 (0.65–0.82)
Did not complete Year12	-	-	-	-	-	-	-	-
Country of birth								
Australia	1.22 (0.98–1.51)	1.31 (1.03–1.66)	1.04 (0.87–1.24)	1.10 (0.90–1.33)	0.89 (0.73–1.09)	0.78 (0.63–0.97)	0.97 (0.86–1.09)	0.88 (0.77–1.00)
Overseas	-	-	-	-	-	-	-	-
Marital status								
Never married	-	-	-	-	-	-	-	-
Widowed, divorced, separated	1.03 (0.57–1.88)	0.73 (0.38–1.41)	1.61 (1.14–2.27)	1.36 (0.91–2.04)	1.09 (0.64–1.86)	0.86 (0.49–1.53)	1.37 (1.08–1.75)	1.35 (1.01–1.80)
Married/de facto	1.32 (1.11–1.57)	1.17 (0.94–1.47)	1.56 (1.23–1.98)	1.57 (1.15–2.15)	0.76 (0.65–0.89)	0.67 (0.54–0.82)	1.00 (0.84–1.20)	0.95 (0.75–1.20)
Employment status								
Currently employed	1.46 (1.17–1.83)	1.48 (1.16–1.89)	0.64 (0.47–0.86)	0.79 (0.56–1.11)	0.94 (0.76–1.16)	1.01 (0.80–1.28)	0.70 (0.58–0.83)	0.83 (0.68–1.02)
Currently unemployed	-	-	-	-	-	-	-	-
Private health insurance								
Yes	1.40 (1.18–1.66)	1.34 (1.11–1.62)	1.39 (1.19–1.62)	1.24 (1.04–1.47)	1.03 (0.88–1.20)	1.02 (0.86–1.20)	1.21 (1.09–1.35)	1.25 (1.11–1.42)
No	-	-	-	-	-	-	-	-
Did experience financial difficulty in past 12 months								
Yes, at least one	1.27 (1.06–1.53)	1.33 (1.08–1.63)	1.10 (0.92–1.30)	1.06 (0.87–1.29)	0.93 (0.79–1.09)	0.82 (0.68–0.98)	0.87 (0.78–0.98)	0.70 (0.62–0.80)
No, none	-	-	-	-	-	-	-	-
Being a father								
One or more children	1.39 (1.15–1.68)	1.39 (1.09–1.77)	1.30 (1.06–1.60)	1.22 (0.93–1.59)	0.87 (0.74–1.03)	1.01 (0.81–1.25)	0.96 (0.82–1.11)	0.93 (0.77–1.13)
No children	-	-	-	-	-	-	-	-
Health and lifestyle factors								
Currently smoking								
Yes	0.90 (0.73–1.11)	0.86 (0.68–1.10)	0.66 (0.55–0.79)	0.66 (0.54–0.82)	1.07 (0.88–1.29)	1.12 (0.90–1.39)	0.82 (0.72–0.94)	0.87 (0.74–1.02)
No	-	-	-	-	-	-	-	-
Drinking								
Harmful consumption	1.01 (0.85–1.20)	1.07 (0.89–1.29)	0.91 (0.78–1.07)	1.02 (0.86–1.21)	0.82 (0.70–0.96)	0.80 (0.68–0.94)	0.77 (0.70–0.86)	0.73 (0.65–0.82)
Non harmful consumption	-	-	-	-	-	-	-	-
Self-rated health								
Very good/excellent	0.86 (0.71–1.03)	1.05 (0.85–1.29)	0.57 (0.49–0.68)	0.68 (0.57–0.83)	0.86 (0.73–1.02)	0.99 (0.82–1.18)	0.79 (0.71–0.88)	1.12 (0.98–1.25)
Good or less	-	-	-	-	-	-	-	-
Being obese								
Yes	1.13 (0.90–1.42)	0.92 (0.72–1.18)	1.59 (1.32–1.91)	1.13 (0.92–1.38)	1.44 (1.19–1.74)	1.42 (1.15–1.74)	1.57 (1.41–1.76)	1.33 (1.17–1.50)
No	-	-	-	-	-	-	-	-
Daily pain self-medication								
Yes	1.98 (1.13–3.47)	1.58 (0.86–2.87)	3.89 (2.59–5.85)	1.91 (1.24–2.94)	1.45 (0.99–2.12)	1.21 (0.81–1.80)	2.34 (1.97–2.77)	1.56 (1.29–1.88)
No	-	-	-	-	-	-	-	-

Logistic Regression Analyses; unweighted data; Total Sample = 13,763, Analytical sample = 9501, omitted cases = 4262; 18–34 years *N* = 3358, 35–55 years *N* = 6143

Among 35 to 55 years old participants, the odds of consultation with a GP were increased with: having regular yearly health check-ups (OR = 7.0, 95 % CI: 5.5–9.0); with an increasing number of heath conditions and was greatest for those with three or more conditions compared with none (OR = 3.2, 95 % CI: 2.0–4.9); with being married (OR = 1.6, 95 % CI: 1.2–2.2); having private health insurance (OR = 1.2, 95 % CI: 1.0–1.5); and taking daily pain medication (OR = 1.9, 95 % CI:1.2–2.9). The odds of consultation with a GP were decreased in this age group with residing in inner regional Australia (OR = 0.8, 95 % CI: 0.6–0.9), but no association was observed for those residing in outer regional Australia. Further, the odds for visiting a GP were reduced for smokers (OR = 0.7, 95 % CI: 0.5–0.8) and rating one’s own health as excellent or very good compared with good or less than good (OR = 0.7, 95 % CI: 0.6–0.8) (Table 1).

### Regular health check-up visits at least once per year

A total of 5167 men (39 %, 95 % CI: 38.3–39.9) reported visiting a family doctor for a general health check at least once per year. There was evidence for a trend towards increasing odds for having at least yearly health check-ups with increasing age (*p* < 0.001) but no significant trend with remoteness of residence was found (*p* = 0.596).

Significant interaction effects were found between age and GP visit in the past 12 months (*p* = 0.003), age and marital status (*p* = 0.041), and age and the number of diagnosed health conditions in the past 12 months (*p* < 0.001). In Table 1 we report the stratified final regression models for regular health check-up visits by age groups.

Among 18 to 34 years old participants, multivariate analysis found that the odds of having regular yearly health check-ups at least once a year were increased with: having visited the GP in the past 12 months (OR = 4.4, 95 % CI: 3.3–5.9); having been diagnosed with two or more health conditions in the past 12 months (OR = 1.8, 95 % CI: 1.3–2.3); and being obese (OR = 1.4, 95 % CI: 1.2–1.7). In this age group, the odds of having yearly health check-ups were decreased for men born in Australia (OR = 0.8, 95 % CI: 0.6–1.0), being married or in a de-facto relationship (OR = 0.7, 95 % CI: 0.5–0.8), having experienced financial difficulties in the past 12 months (OR = 0.8, 95 % CI: 0.7–1.0), and engaging in harmful drinking (OR = 0.8, 95 % CI: 0.7–0.9).

Among 35 to 55 years old participants, the odds for regular check-ups were increased with: having visited the GP in the past 12 months (OR = 7.0, 95 % CI: 5.5–9.0); experiencing an increasing number of heath conditions and was greatest for those with three or more conditions compared with none (OR = 3.1, 95 % CI: 2.5–3.8); being widowed, divorced or separated (OR = 1.4, 95 % CI: 1.0–1.8); having private health insurance (OR = 1.3, 95 % CI: 1.1–1.4); being obese (OR = 1.3, 95 % CI: 1.2–1.5); and taking daily pain medication (OR = 1.6, 95 % CI: 1.3–1.9). A decrease in odds for having regular check-ups was found for those who completed secondary school (OR = 0.7, 95 % CI: 0.7–0.8), experienced financial difficulties (OR = 0.7, 95 % CI: 0.6–0.8), and for those who engaged in harmful drinking (OR = 0.7, 95 % CI: 0.7–0.8).

Overall, 4262 (31 %) men were excluded from the multivariate analysis because of missing data. Those excluded from the analysis were more likely to be younger (*p* < 0.01), less likely to be married (*p* < 0.01), less likely to have completed secondary school education (*p* < 0.01) and more likely to have been born overseas (*p* < 0.01). There was no difference in remoteness of residence (*p* = 0.32).

## Discussion

The findings of our study are consistent with the Australian data on male consultations with the GP - BEACH data shows 80 % of males in the population saw a GP at least once in 2013 to 2014. While this suggests that a high proportion of males are seeing a GP on a regular or yearly bases it must be stressed that these proportions fall short compared to women’s rates of GP visits [6]. This paper highlights some of the complexities that research into men’s health care utilisation and help seeking faces.

We found that 81 % of participants reported having consulted a GP in the last 12 months with increasing odds of visiting a GP as age increases and decreasing odds as remoteness of residence increases. We also found that only 39 % of men had regular health check-ups and while the odds increased with age, there was no association with the remoteness of residence. Our analyses show that a person’s general health status affects the odds of visiting a GP. Men with very good to excellent self-rated health were less likely to have visited a GP in the last 12 months but those taking pain medication on a daily basis and those who were diagnosed with one or more health conditions were more likely. The magnitude of effects was greater for older than younger men. Across men of all ages, having private health insurance increased the odds for a GP visit in the past 12 months.

Socio-demographic factors showed fewer significant effects on the odds for visiting a GP in older men compared with younger men. We found that those living outside of a major city were less likely to attend a GP in the last 12 months. This raises issues of equity in terms of healthcare access for those living in rural and regional Australia. People living in rural areas have less choice of healthcare services available and they are more likely to have longer travel distances to attend these services mostly without access to public transport [50–52]. Medical workforce shortages in rural areas makes access to health services even more challenging [53]. Rural health services have to provide care to a more dispersed population than urban services while at the same time, are often smaller, less resourced and face additional expenses associated with distance [54].

Health and lifestyle factors showed greater relevance among older men with those smoking and those with excellent to good self-rated health attending the GP less often. Of concern was that smokers were less likely to have consulted a GP in the last 12 months which means that the opportunity to influence lifestyle choices or even monitor health risks in this population group is diminished. It has been well established that smoking is associated with lower socio-economic status [55, 56], but it is a concern that even after adjusting for socio-economic variables such as education, remoteness and financial difficulties, our study shows that older smokers are less engaged with the healthcare system. The factors that were most strongly related to past GP visits seem to measure ill health (e.g., taking daily pain-medication, having been diagnosed with health conditions over the past 12 months and rating own health less positive). This suggests that men may see a GP as particular health conditions present or on a needs basis, rather than planning their visit in advance.

In contrast to GP visits, the odds of engaging in regular health check-ups were reduced by having finished secondary school, being born in Australia, having experienced financial difficulties or being married. In addition to factors measuring ill health (i.e. taking daily pain medication and number of diagnosed health conditions) the health risk factor of being obese increased the odds for regular health check-ups. While this provides an opportunity for GPs to discuss healthy lifestyle choices with at risk men, the overall low number of men choosing to see a doctor for a general health check-up is reducing the significance of this opportunity. Further, engaging in harmful alcohol consumption decreased the odds for regular health check-ups meaning a reduced opportunity for intervention by a doctor or medical professional.

The majority of men (61 %) did not engage in regular health check-up visits, which we believe represents a missed opportunity for preventative health care discussions. Health check-ups have been demonstrated to improve the frequency of preventive care and support regular discussions on changing health behaviour in middle age [57, 58] and have also been found to improve the quality of preventative care [59]. Further, adults who received ongoing care from regular visits to the GP are found to be more likely to receive the preventive services as recommended by policy guidelines [18]. Lower consultation rates may therefore translate into lost opportunities to detect and intervene with problems early and this is where men may be missing out compared to women [19].

It is possible that existing funding mechanisms in Australian general practice are deterrents against routine health checks [60]. For example, among those aged 45 to 49 years with documented risk factors for chronic disease, GPs can only claim one cardiovascular health check consultation over this five year period [17, 61]. Providing GPs with more options to initiate preventive health care discussions with patients could increase health care utilisation among men, especially for problems where stigma is perceived or experienced (i.e. mental health problems) [14]. Regular contact with a medical professional can help normalize medical consultations for men allowing them to build trust with medical professionals and be more inclined to be proactive in their health management or seek help when issues arise. Failing to seek help for symptoms can lead to delayed diagnosis and treatment and increase the long term burden on the healthcare system [62]. However, it should be acknowledged, that the benefits of increased health care utilisation for preventive health checks do need to be balanced against the potentially increased costs of screening tests and other medical investigations. Future analyses of the Ten to Men cohort study will involve linkage with government health care utilisation records (Medicare) providing further opportunities to explore associations between health care utilisation and health outcomes.

There are several limitations of this analysis which must be considered when interpreting the data. Firstly, we did not include social or emotional and cognitive psychological factors as predictors (e.g., masculine norms, gender roles, or attitudes to help seeking). In the context of health services utilisation it is plausible that attitudes to health and help seeking play an important role [27, 28]. Visiting the doctor for regular health check-ups might see a different sub-group of men and most likely those men who show initiative in looking after their health and are engaged with their health and health services in general. In contrast, GP visits in the last 12 months as an outcome measure might capture those men who saw a doctor because of a specific health care need (i.e. illness or injury). However, investigating the role of attitudes and social roles requires more complex analysis which was beyond the scope of this paper. Secondly, the measurement of health care utilisation used in this paper is based on self-report which is likely to be subject to recall bias. At the time of analysis we did not have access to participants’ health records including types of services accessed, diagnostic tests performed and medications prescribed. These data would add crucial information to further our understanding of this field and address any bias caused by self-report.

The strengths of this analysis are that a range of socio-demographic and health-related variables were available allowing for a comprehensive analysis of potential associates of health care utilisation in men. Any contact with a GP offers the opportunity for diagnosis and intervention. However, more research is needed to better understand differences in sub-groups of men in terms of what motivates their help seeking behaviour. For example, a profile analysis of those men who do not use any health services could provide useful information to guide interventions or health promotion strategies to reach them for healthcare.

## Conclusion

Males’ healthcare utilisation is varied and is greatly influenced by age and the interaction between age and general health status. The proportion of men having regular health check-ups is generally much lower than the proportion of men visiting a GP in the past. Further, older men engage more often in regular health check-ups which might be driven by a need to monitor existing health conditions. The differences between the two measures of health care utilisation indicate that past GP visits and regular check-up visits are driven by different contextual and individual behavioural factors. Our findings suggest that men may see a GP only as needed (e.g., for acute illness or injury) and may not plan their visit on a regular basis, and that contextual factors are of higher relevance in deciding on whether or not to see a GP. More research is needed to better understand what social, behavioural and health factors influence different aspects of medical help seeking in men. However, lower check-up rates may translate into lost opportunities to detect and intervene with problems early and this is where men may be missing on receiving the preventative health care when needed.
